# Self-injury in adolescents: expression of subjective discontent in contemporary society

**DOI:** 10.1192/j.eurpsy.2022.1129

**Published:** 2022-09-01

**Authors:** A. Dörr Alamos, S. Viani

**Affiliations:** 1 University oy Chile, Faculty Of Medicine, Metropolitana, Chile; 2 University of Chile, Faculty Of Medicine, Santiago, Chile

**Keywords:** adolescence, self-injuries, identity, adolescence, self-injuries, characteristics of current society, identity

## Abstract

**Introduction:**

This research aims to enrich the reflection on the current affairs and is an invitation to think about worrying phenomena in youngsters: self-injuries or cuts. A focused study was conducted on twenty young people

**Objectives:**

This ris a reflection oabout worrying phenomena in youngsters: self-injuries or cuts. We try to figure out the underlying cause of such behaviour

**Methods:**

Through a qualitative methodology, using clinical interview and questionnaire, we try to find the constitutive elements of self-injuries or cuts, considering three levels of analysis: Sociocultural, individual, and family changes. We examined twenty cases

**Results:**

It appeared in the analysis that one of the triggers of the cutting phenomenon was related to experiences of rejection of significant figures, which is lived by the young person as a situation of abandonment that generates high amounts of anguish. This distressing experience generates a sensation of lack of control, appearing as unregulated affections, which they are unable to handle or manage. The intense anxiety is carried through the body, being an act of attack to the body, provokes physical pain an emotional relief

**Conclusions:**

Young people’s subjective perception of the maternal figure is that of a dominant, controlling and demanding personality, which hinders an adequate differentiation process. With respect to the description that the young people make of themselves, we see that they refer to a marked emotional lability and the presence of dysthymic experiences. It appeared in the analysis that one of the triggers of the cutting phenomenon was related to experiences of rejection or separation of significant figures.

**Disclosure:**

No significant relationships.

